# „Schwierige“ Patient:innen – Vestibularisdiagnostik unter erschwerten Bedingungen: Teil 2: Schwierige Aspekte der apparativen Vestibularisprüfung

**DOI:** 10.1007/s00106-023-01401-y

**Published:** 2024-01-23

**Authors:** Julia Dlugaiczyk

**Affiliations:** Klinik für Ohren‑, Nasen‑, Hals- und Gesichtschirurgie & Interdisziplinäres Zentrum für Schwindel und neurologische Sehstörungen, Universitätsspital Zürich (USZ), Universität Zürich (UZH), Rämistrasse 100, 8091 Zürich, Schweiz

**Keywords:** Kopfimpulstest, Vestibulär evozierte myogene Potenziale, Vibrationsnystagmus, Kalorische Tests, Videookulographie, Head impulse test, Vestibular evoked myogenic potentials, Vibration nystagmus, Caloric tests, Videooculography

## Abstract

Patient:innen mit dem Leitsymptom „Schwindel“ stellen häufig eine diagnostische Herausforderung für die betreuenden Hals-Nasen-Ohren-Ärzt:innen dar. Während im ersten Teil dieser Fortbildungsreihe der Fokus auf der Anamnese und klinisch-neurootologischen Untersuchung lag, behandelt der vorliegende zweite Teil wichtige Aspekte der „schwierigen“ apparativen Vestibularisprüfung, insbesondere: Indikationsstellung, Lösungsansätze bei eingeschränkter Kooperationsfähigkeit der Patient:innen, Auswahl der vestibulären Tests in Abhängigkeit von Komorbiditäten, Interpretation von diskrepanten Befunden aus einzelnen Teiluntersuchungen. Des Weiteren wird dargelegt, welche Schlussfolgerungen aus einer normwertigen Vestibularisprüfung gezogen werden können (und welche nicht) und wie dieses Ergebnis den Patient:innen erläutert werden kann.

## Abstract (en)

Patients with the cardinal symptoms “vertigo” or “dizziness” may be a real challenge for the treating otorhinolaryngologist. While the first part of this educational series was focused on history taking and bedside neurotological examination, the present paper is devoted to difficult aspects of vestibular laboratory testing, including getting the indication right, what to do if my patient is not able to fully cooperate during the tests, how to choose the adequate diagnostic procedure depending on the patient’s comorbidities, how to interpret discordant results of various tests. Finally the paper addresses which conclusions can be drawn (and cannot be drawn) from normal findings in vestibular testing and how to communicate this result to the dizzy patient.

## Lernziele

Nach Absolvieren dieser Fortbildungseinheit …

- können Sie mindestens 3 Indikationen für die apparative Vestibularisdiagnostik benennen,
- sind Sie in der Lage, verschiedene Techniken anzuwenden, um die Kooperation Ihrer Patient:innen während der Untersuchungen zu verbessern,
- können Sie die Teiluntersuchungen einer apparativen Vestibularisprüfung in Abhängigkeit von den Komorbiditäten der Patient:innen auswählen,
- kennen Sie mögliche Ursachen für diskordante Befunde zwischen einzelnen vestibulären Tests,
- können Sie Ihren Patient:innen den Stellenwert von normwertigen Befunden der Vestibularisprüfung erläutern.

## Hinführung zum Thema

Nachdem in Teil 1 dieser CME-Fortbildungsreihe zusammen mit den „schwierigen“ Schwindelpatient:innen die Anamneseerhebung und klinisch-neurootologische Untersuchung gemeistert wurde [1], wartet mit der apparativen Vestibularisprüfung schon die nächste Herausforderung. **Durchführung**

Durchführung,

Auswertung und **Befundbesprechung**

Befundbesprechung

sind häufig mit einem Gefühl der Frustration und Verzweiflung verbunden, sowohl aufseiten des Untersuchers („Jetzt lassen Sie doch mal die Augen auf.“) als auch aufseiten des Untersuchten („Jetzt haben Sie auch wieder nichts gefunden.“). Das muss nicht so sein! Der vorliegende Teil der Fortbildungsreihe behandelt schwierige Aspekte der apparativen Vestibularisdiagnostik im **nichtakuten Setting**

Nichtakutes Setting.

Wie bereits im ersten Teil geht es hier nicht um eine Sammlung von „Tipps und Tricks“ oder Patentlösungen, sondern um Anregungen aus der Praxis für die Praxis.

Im Folgenden werden die Begriffe „Vestibularisdiagnostik“ und „apparative Vestibularisdiagnostik“ synonym verwendet. Eine Auswahl praxisrelevanter Übersichtsarbeiten zu diesem Thema findet sich in Tab. 1. Dabei sei insbesondere auf die informativen Gegenüberstellungen einzelner vestibulärer Testverfahren in Tab. 1 der Arbeit von Zuniga und Adams [2] und in Tab. 3 der Arbeit von Walther [3] hingewiesen.

**Table 1:** 

Vestibuläre Untersuchungsmethode	Referenzen
Allgemeine Übersichtsarbeiten	[2, 3, 4, 5, 6, 7]
Video-Kopfimpulstest (vHIT)	[8, 9, 10]
Vestibulär evozierte myogene Potenziale (VEMP)	[11, 12, 13]
Vibrationsinduzierter Nystagmus (VIN)	[12, 14, 15, 16]

## Indikationsstellung

Bevor es um die schwierigen Aspekte der Vestibularisdiagnostik im Detail geht, lohnt sich eine Betrachtung der Fragen, was diese Untersuchungstechniken leisten können und wo ihre Grenzen liegen. Die Vestibularisdiagnostik sollte *nicht* als eine Screening-Untersuchung zur Suche peripher- und/oder zentral-vestibulärer Störungen verwendet werden, sondern sie sollte immer mit einer **konkreten Fragestellung**

Konkrete Fragestellung

verknüpft sein [2]. Vor jeder Diagnostik sollten sich die indizierenden Ärzt:innen die folgenden Fragen stellen:

- Welche konkrete Frage soll mit der Untersuchung beantwortet werden?
- Welche Untersuchung ist hierfür am besten geeignet?
- Ändert sich durch das Ergebnis der Untersuchung die Diagnose oder die geplante Therapie?

### Was kann Vestibularisdiagnostik?

Die apparative Vestibularisdiagnostik ermöglicht folgende Aussagen über eine vestibuläre Störung [2]:

- Lokalisation,
- Quantifizierung des Ausmaßes eines vestibulären Defizits,
- Beurteilung der Kompensation eines vestibulären Defizits,
- Beurteilung der Integrität der vestibulookulären und -spinalen Bahnen.

Aussagen zur Lokalisation können z. B. sein: peripher vs. zentral, rechts vs. links oder die Zuordnung zu den einzelnen Rezeptorsubtypen (Bogengänge, Otolithenorgane). Hinsichtlich der Quantifizierung des Ausmaßes eines vestibulären Defizits eignen sich z. B. Befunde wie eine Gain-Reduktion im Video-Kopfimpulstest (vHIT) oder die Asymmetrie-Ratio bei den vestibulär evozierten myogenen Potenzialen (VEMP). Zur Beurteilung der Kompensation eines vestibulären Defizits kann das Vorhandensein eines Spontan‑, Provokations- und/oder Lagenystagmus als Zeichen einer unkompensierten Störung im Bereich der Bogengänge dienen. Die Verkippung der subjektiven visuellen Vertikalen (SVV) kann Zeichen einer unkompensierten Störung der Otolithenorgane bzw. -bahnen sein. Eine Beurteilung der Integrität der vestibulookulären Bahnen kann z. B. mit dem vHIT oder der Videookulographie (VOG) erfolgen, und für die vestibulospinalen Bahnen eignet sich z. B. die Posturographie.

### Was kann Vestibularisdiagnostik nicht?

- Vestibuläre Diagnosen stellen,
- Krankheitsempfinden, Einschränkungen im Alltag und Einfluss auf die Lebensqualität erfassen.

Die Vestibularisdiagnostik ist keine „Juke Box“, in die man oben Befunde eingibt und aus der unten eine Diagnose herauskommt [2, 17]. Die Diagnose vestibulärer Störungen entsteht immer aus der Synthese von Anamnese, klinisch-neurootologischer Untersuchung und ggf. apparativer Vestibularisdiagnostik, wobei der prädiktive Wert der Anamnese bei bis zu 80 % liegt [2] (s. auch Teil 1 der Fortbildungsreihe). Des Weiteren kann die apparative Vestibularisdiagnostik nicht das subjektive Krankheitsempfinden, die resultierenden Einschränkungen im Alltag und den Einfluss der Schwindelbeschwerden auf die Lebensqualität der Patient:innen erfassen. Die Anamnese sowie **Fragebögen**

Fragebögen

zur Lebensqualität und zur **patient:innenzentrierten Ergebnisbewertung**

Patient:innenzentrierte Ergebnisbewertung

(PROMs, „patient-related outcome measures“) stellen hier geeignete Werkzeuge dar [18, 19]. Weitere Details finden sich in Teil 1 („Herausforderung 5“) [1].

#### Merke

Vestibuläre Erkrankungen werden immer in der Gesamtschau von Anamnese, klinisch-neurootologischer Untersuchung und ggf. apparativer Vestibularisprüfung diagnostiziert.

## Eingeschränkte Kooperation der Patient:innen

### Maßnahmen zur Förderung der Kooperation

„Nun lassen Sie doch mal die Augen auf!“ Hand aufs Herz: Ist Ihnen dieser Satz auch schon mal in einem unbedachten Moment herausgerutscht? Das Dilemma ist offensichtlich. Die meisten apparativen vestibulären Tests basieren auf einer Aufzeichnung von Augenbewegungen zur Untersuchung des vestibulookulären Reflexes (VOR), z. B. VOG, Kalorik, Drehstuhlpendelung, vHIT [2]. Nur ist dieser Zusammenhang den meisten der Patient:innen nicht bewusst: Aus ihrer Perspektive sind die Untersuchungen oft unangenehm, sie provozieren Übelkeit und lösen teils den gleichen Schwindel aus, den sie von invalidisierenden Attacken kennen. Der **reflektorische Augenschluss**

Reflektorischer Augenschluss

ist daher eher ein Ausdruck von **Angst**

Angst

und Stress als von mangelnder Kooperation.

Daher ist es essenziell, den Patient:innen vor der Untersuchung die zugrunde liegende Physiologie in einfachen Worten zu erklären und die zentrale Bedeutung ihrer Mitarbeit zu betonen, z. B. „Ihre Augen sind für mich das Fenster zu Ihrem Gleichgewichtsorgan. Ich kann Ihr Innenohr nicht sehen, weil es tief im Knochen der Schädelbasis liegt. Ihre Augenbewegungen erlauben es mir aber, einen Einblick in die Funktion Ihres Gleichgewichtssystems zu gewinnen. Deshalb ist es für mich ganz wichtig, dass Sie versuchen, die Augen eine Minute lang offen zu halten. Schaffen Sie das?“

Die Frage nach **spezifischen Ängsten**

Spezifische Ängsten

(z. B. Klaustrophobie) oder **negativen Vorerfahrungen**

Negative Vorerfahrungen

mit Vestibularisdiagnostik (z. B. Erbrechen bei der Kalorik) ermöglicht es dem Untersuchenden, hierauf gezielt einzugehen und die Untersuchungen darauf abzustimmen. Bei Klaustrophobie ist es z. B. möglich, die VOG im abgedunkelten Raum, jedoch ohne Aufsatz der Verdunklungsklappe an der Videobrille durchzuführen. Berichten die Patient:innen über eine heftige vegetative Reaktion bei einer kalorischen Prüfung in der Vergangenheit, ist dies ein klares Indiz für eine kalorische Erregbarkeit der Vestibularorgane. Daher kann auf eine erneute Kalorik getrost verzichtet werden (für Alternativen s. Abschnitt „Untersuchung der Bogengangsfunktion“).

Bei Patient:innen mit eingeschränkter kognitiver Funktion oder bei kleinen Kindern lässt sich beim vHIT oft beobachten, dass die Augen in Richtung der Kopfdrehung abweichen und nicht auf das Blickziel fixiert bleiben. Hier stellen die **SHIMPs**

**Figure 1:**
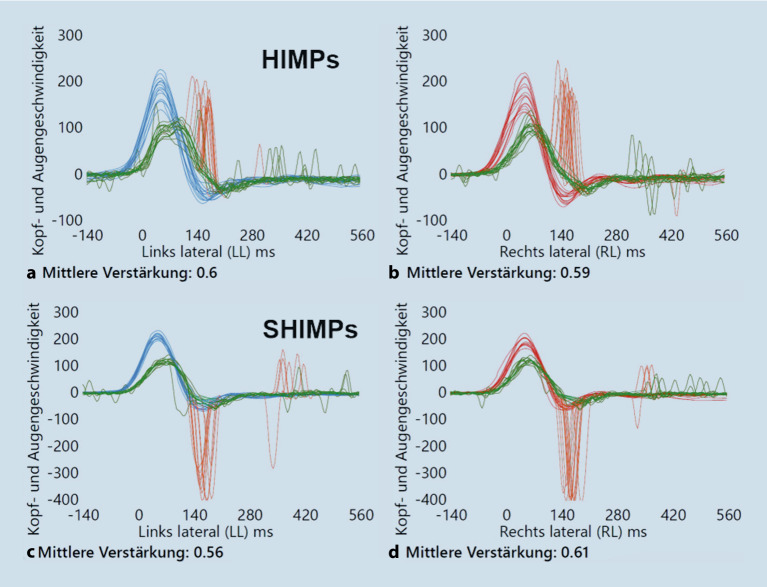

SHIMPs

(„suppression of head impulses“) eine wertvolle Alternative dar (Abb. 1). Die Patient:innen sollen bei dieser Variante des vHIT explizit einem bewegten Blickziel folgen, was eine deutlich intuitivere Aufgabe darstellt [8, 20, 21]. Durch die veränderte Aufgabenstellung (HIMPs: Fixation eines stationären Blickziels; SHIMPs: Fixation eines bewegten Blickziels) ändert sich die Konfiguration der Sakkaden, während die Gain-Werte („mittlere Verstärkung“ in Abb. 1) für die beiden lateralen Bogengänge weitgehend unverändert bleiben.

Zusammenfassend kommt der **Auswahl**

Auswahl

der apparativen Tests und ihrer **Reihenfolge**

Reihenfolge

abhängig von der Fragestellung eine entscheidende Rolle zu. Wie bei der klinischen Untersuchung sollten die „schwindelerregenden“ Tests (z. B. Kalorik) erst am Ende des Untersuchungsgangs durchgeführt werden [1]. Ist die Untersuchungszeit der limitierende Faktor (z. B. aufgrund von Konzentrationsschwierigkeiten, Rückenschmerzen, Angst), sollte mit denjenigen Untersuchungen begonnen werden, von denen die wichtigsten Aussagen erwartet werden. Die Basis hierfür ist wieder eine sorgfältig erhobene Anamnese mit klar formulierten Fragen an die Vestibularisprüfung.

#### Merke

Besser wenige Untersuchungen mit guter Qualität als viele Untersuchungen mit schlechter Qualität.

Es hat sich auch bewährt, den Patient:innen **Pausen**

Pausen

bei der Untersuchung anzubieten oder **Stopp-Signale**

Stopp-Signale

zu vereinbaren, welche zu einer sofortigen Unterbrechung der Untersuchung führen. Schaffen es die Untersucher:innen, ihren Patient:innen ein Gefühl der Sicherheit und der Selbstwirksamkeit zu vermitteln, nimmt auch die **Kooperationsbereitschaft**

Kooperationsbereitschaft

in den meisten Fällen zu.

Abschließend sei noch die zentrale Bedeutung der Zusammenarbeit zwischen Patient:innen, Ärzt:innen und dem **technischen Personal**

Technisches Personal

erwähnt. Eine erfahrene medizinisch-technische Fachkraft kann die Kooperationsfähigkeit und -bereitschaft der Patient:innen häufig gut einschätzen, insbesondere Situationen, in denen es nur einen einzigen Versuch gibt, die Untersuchung durchzuführen. Sollte die Fachkraft Sie in einer solchen Situation als Arzt/Ärztin hinzurufen, zögern Sie nicht, dem nachzukommen. Es könnte Ihre einzige Möglichkeit sein, das Untersuchungsergebnis (z. B. ein bestimmtes Nystagmusmuster) live zu sehen, welches sich bei eingeschränkter Kooperation nur schwer aufzeichnen lässt.

### Alternativen bei Undurchführbarkeit von Tests

Und wenn sich gar keine Augenbewegungen aufzeichnen lassen? Trotz aller Mühe und Hingabe gibt es Patient:innen, bei denen die Videoaufzeichnung von Augenbewegungen zur Beurteilung der vestibulären Funktion nicht möglich oder nicht sinnvoll ist. Hierzu zählen z. B. (kleine) Kinder, Patient:innen mit kognitiven Einschränkungen oder mit einem kongenitalen/infantilen Nystagmus [22]. Hier bieten sich als Alternative Tests an, welche nicht auf die Aufzeichnung von Augenbewegungen angewiesen sind, z. B. **VEMPs**

VEMPs.

So lässt sich zumindest eine **seitengetrennte Aussage**

Seitengetrennte Aussage

über die Funktion des N. vestibularis superior und inferior treffen [11].

VEMPs eigenen sich insbesondere wegen der **kurzen Registrierungsdauer**

Kurze Registrierungsdauer

(20 s bei 100 Stimuli und 5 Stimuli/s) sehr gut für Kinder und Patient:innen mit kognitiven Einschränkungen. Die Kopfdrehung bei den zervikalen VEMPs (cVEMPs) und der Aufblick bei den okulären VEMPs (oVEMPs) kann bei diesen Patient:innengruppen durch das Abspielen von Videofilmen in der gewünschten Blickrichtung erleichtert werden [23].

## Einfluss von Komorbiditäten auf die Wahl der Untersuchungsmethoden

Neben dem kongenitalen/infantilen Nystagmus wird die Auswahl der geeigneten Tests durch weitere Komorbiditäten beeinflusst. Eine Zusammenfassung der möglichen Lösungsansätze findet sich in Tab. 2.

**Table 2:** 

Problem	Mögliche Lösung
vHIT: eingeschränkte Fixation des stationären Blickziels	Verwenden eines bewegten Blickziels (SHIMPs)
Keine kalorische Prüfung möglich (z. B. wegen vegetativer Reaktion, Radikalhöhle, Trommelfellperforation)	vHIT: Quantifizierung der Funktion aller 6 Bogengänge
	VIN: Tonusdifferenz der beiden horizontalen Bogengänge (unkompensiert und kompensiert)
	Drehstuhlpendelung: Tonusdifferenz der beiden horizontalen Bogengänge (nur unkompensiert)
Keine Registrierung des VOR möglich oder sinnvoll (z. B. mangelnde Kooperation, infantiler Nystagmus)	Vom VOR unabhängige Untersuchungen verwenden, z. B. VEMPs
VEMPs: – Schallleitungsschwerhörigkeit – Lärmtrauma/Tinnitus/Hyperakusis	Knochenleitungs- statt Luftleitungs-VEMPs verwenden

*SHIMPs *„suppression of head impulses“, *VEMPs *„vestibular evoked myogenic potentials“, *vHIT *Video-Kopfimpulstest, *VOR* vestibulookulärer Reflex, *VIN* vibrationsinduzierter Nystagmus

### Untersuchung der Bogengangsfunktion

Das Vorliegen einer **Radikalhöhle**

Radikalhöhle

oder einer **Trommelfellperforation**

Trommelfellperforation

ist eine Kontraindikation für eine kalorische Prüfung mit Wasser [4]. Alternativ steht hier der **vHIT**

vHIT

zur Beurteilung der horizontalen – und zusätzlich vertikalen – Bogengänge zur Verfügung [8]. Sollte auch diese Untersuchung nicht möglich sein, z. B. aufgrund von Einschränkungen der Kopfbeweglichkeit, gibt es weitere Alternativen, um zumindest eine **Tonusdifferenz**

Tonusdifferenz

zwischen den beiden horizontalen Bogengängen zu bestimmen. Zum einen besteht die Möglichkeit der **Drehstuhlpendelung**

Drehstuhlpendelung.

Bei einer unkompensierten einseitigen Unterfunktion des horizontalen Bogengangs zeigt sich hier ein Überwiegen der Nystagmen in Richtung des gesunden Ohrs (höherer Tonus). Der Nachteil der Drehstuhluntersuchung besteht jedoch darin, dass es mit zunehmender zentraler Kompensation der Störung zu einem Ausgleich des Richtungsüberwiegens kommt, sodass ein Defizit auf Ebene der Bogengänge im Verlauf häufig nicht mehr nachweisbar ist [2].

Der **vibrationsinduzierte Nystagmus**

**Figure 2:**
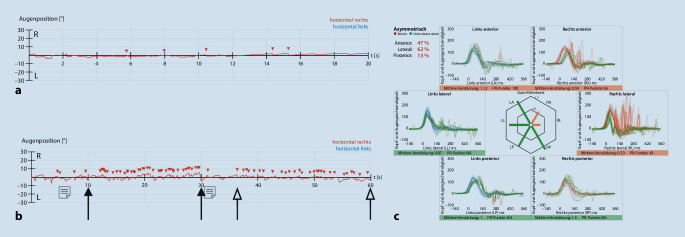

Vibrationsinduzierter Nystagmus

(VIN) mit Applikation eines 100-Hz-Stimulus am Mastoid stellt eine schnelle und gut tolerierte Alternative dar, um am Patientenbett eine Tonusdifferenz der horizontalen Bogengänge zu detektieren (Abb. 2). Selbst eine lange bestehende (teil)**kompensierte Unterfunktion**

Kompensierte Unterfunktion

kann hierdurch demaskiert werden (s. [14, 24] für die zugrunde liegende (Patho‑)Physiologie und [12, 15, 16] für die klinische Anwendung). Bei dem Patienten in Abb. 2 wurden Spontan- und vibrationsinduzierter Nystagmus mittels VOG im Dunkeln ohne Fixation aufgezeichnet. Ein **Spontannystagmus**

**Figure 3:**
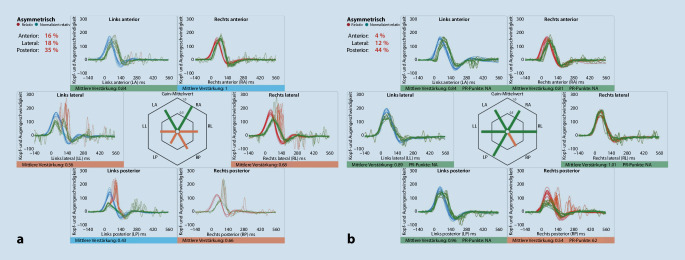

Spontannystagmus

ist nicht mehr vorhanden, als Zeichen der zentral-vestibulären Kompensation (Abb. 2a). Der Nachweis eines VIN nach links während Applikation eines 100-Hz-Vibrationsstimulus auf das linke Mastoid und das rechte Mastoid demaskiert eine weiterhin bestehende relative Unterfunktion des rechten horizontalen Bogengangs (Abb. 2b). Im vHIT zeigt sich eine Unterfunktion des lateralen und anterioren Bogengangs rechts, passend zu einer Neuritis vestibularis superior rechts (Abb. 2c). Weitere pathognomonische vHIT-Muster finden sich in Abb. 3.

### Untersuchung der Otolithenfunktion

Liegt eine **Schallleitungsschwerhörigkeit**

Schallleitungsschwerhörigkeit

im Bereich des Mittelohrs vor, so sollte für die Registrierung der VEMPs in jedem Fall ein Knochenleitungs- und kein Luftleitungsstimulus verwendet werden. Bereits ein Air-Bone-Gap von 10 dB reduziert die im Vestibulum ankommende Schallenergie so stark, dass die VEMP-Antworten reduziert sind oder ganz fehlen. Im Gegensatz dazu hat eine reine **Sensorineurale Schwerhörigkeit**

sensorineurale Schwerhörigkeit

keinen Einfluss auf das VEMP-Ergebnis, da es sich hier um rein vestibuläre – und nicht cochleäre – Reizantworten handelt [11].

#### Merke

Vor jeder Messung von Luftleitungs-VEMPs sollte ein Reintonaudiogramm mit Luft- und Knochenleitung durchgeführt werden.

Besonders wichtig ist dieser Aspekt bei Patient:innen, bei denen aufgrund einer hochgradigen Schwerhörigkeit/Taubheit eine evtl. vorhandene Schallleitungskomponente aus technischen Gründen nicht bestimmt werden kann (z. B. nach Cochleaimplantation). Hier sollte generell eine Messung mit **Knochenleitungsstimuli**

Knochenleitungsstimuli

bevorzugt werden, um keine falsch-negativen VEMP-Resultate zu erhalten [26].

Des Weiteren sollte berücksichtigt werden, dass die überschwelligen akustischen Stimuli ein **Lärmtrauma**

Lärmtrauma

auslösen können. Besondere Vorsicht ist hier geboten bei Patient:innen mit Tinnitus, Hyperakusis oder einem kürzlich stattgehabten Hörsturz/Lärmtrauma. In diesen Fällen sind Knochenleitungs- gegenüber Luftleitungsstimuli zu bevorzugen. Stehen keine Knochenleitungsreize zur Verfügung, sollten folgende Punkte beachtet werden [27]:

- Keine zusätzlichen überschwelligen audiologischen Untersuchungen am gleichen Tag durchführen (z. B. Hirnstammaudiometrie, Stapediusreflexe).
- Expositionszeit so kurz wie möglich halten, insbesondere: Messung beenden, wenn sich durch zusätzliche Stimuli keine Veränderungen mehr im gemittelten Potenzial ergeben.
- Falls die Patient:innen während der Messung neu aufgetretene oder akut verstärkte Ohrsymptome angeben, wie Hörminderung, Tinnitus, Schwindel, Otalgie: Messung beenden und die Patient:innen nachbeobachten.

#### Merke

Fragen Sie Ihre Patient:innen vor der Durchführung von Luftleitungs-VEMPs nach Risikofaktoren für ein Lärmtrauma.

## Auswertung

### Diskordante Befunde

Nachdem die einzelnen Untersuchungen der apparativen Vestibularisprüfung erfolgt sind, wartet die nächste Herausforderung. Die beurteilenden Ärzt:innen sehen sich mit einer Fülle an Einzelbefunden konfrontiert, welche oft nicht „zusammenpassen“. Anstatt sich darüber zu ärgern und Ergebnisse als Artefakte oder „nicht plausibel“ abzutun, lohnt es sich vielmehr zu überlegen, warum die Befunde diskordant sein könnten. Hieraus ergeben sich oft wichtige Hinweise für die Diagnosestellung.

#### Mehrere Diagnosen

Eine nützliche Frage in diesem Zusammenhang ist: Lassen sich alle Befunde durch eine einzige zugrunde liegende vestibuläre Störung erklären?

In diesem Zusammenhang sollte man sich immer wieder in Erinnerung rufen, dass bei 16–50 % der Patient:innen mit dem Leitsymptom „Schwindel“ zumindest 2 verschiedene vestibuläre Diagnosen vorliegen [1], z. B. ein benigner paroxysmaler Lagerungsschwindel (BPLS) und eine chronische peripher-vestibuläre Unterfunktion der gleichen Seite im Fall eines postneuritischen BPLS des rechten posterioren Bogengangs. Diese Patient:innen können parallel einen Kopfschüttelnystagmus nach links als Zeichen einer inkomplett kompensierten peripher-vestibulären Unterfunktion rechts und einen rotatorischen Lagerungsnystagmus nach rechts mit Upbeat-Komponente als Zeichen für den posterioren BPLS rechts aufweisen. Eine Übersicht über häufige simultan auftretende vestibuläre Diagnosen findet sich in Tab. 1 der Arbeit von Fife [28].

##### Merke

Bei 16–50 % der Patient:innen mit dem Leitsymptom „Schwindel“ liegen zumindest 2 verschiedene vestibuläre Diagnosen vor.

#### Stellenwert einzelner Befunde

Nicht jeder einzelne Nystagmus in der VOG hat eine pathologische Relevanz. Per definitionem ist ein Nystagmus durch eine repetitive Abfolge von unwillkürlichen Augenbewegungen gekennzeichnet [29]. Daher sollten in der VOG erst mehrere **aufeinanderfolgende Nystagmen**

Aufeinanderfolgende Nystagmen

mit einer Geschwindigkeit der langsamen Phase (GLP) von mindestens 2–3°/s als potenziell pathologisch gewertet werden (abhängig von der Gesamtschau aus Anamnese und Befunden) [2, 16]. Zudem ist bei der Auswertung zu beachten, dass Nystagmen und andere Augenbewegungsstörungen auch als Nebenwirkungen von Medikamenten und Genussmitteln/Drogen auftreten können (Übersichten bei [30, 31]). Bei **Rauchern**

Raucher

lässt sich z. B. häufig ein **Upbeat-Nystagmus**

Upbeat-Nystagmus

ohne pathologische Relevanz beobachten [32].

##### Merke

Führen Sie im Rahmen der Vestibularisdiagnostik eine Genussmittel- und Medikamentenanamnese durch.

Ferner lassen sich beim vHIT häufig **Mikrosakkaden**

Mikrosakkaden

(Spitzengeschwindigkeit etwa 100°/s) bei normalen Gainwerten beobachten. Diese besitzen i. d. R. keine pathologische Relevanz. Allein der Blick von einem Ende des Fixationspunkts zum anderen kann diese kleinen Augenbewegungen auslösen [8].

Ein guter Rat eines ehemaligen Kollegen lautete: „Halt die Befunde bei der Auswertung lieber etwas weiter weg. Dann siehst Du eher, wo das Problem liegt.“ In der Tat hilft gerade in diesem Metier der Blick aufs große Ganze und das Erkennen von **typischen Befundkonstellationen**

Typische Befundkonstellationen

mehr als die Analyse eines jeden einzelnen Nystagmus in der VOG oder jeder einzelnen Mikrosakkade im vHIT. Die Abb. 2c und 3 zeigen exemplarisch typische pathognomonische Muster von vHIT-Befunden. Im Gegensatz zur Neuritis vestibularis superior mit einer Unterfunktion des horizontalen und des anterioren Bogenganges (Abb. 2c) ist bei der Neuritis vestibularis inf. in Abb. 3b nur der posteriore Bogengang von einer Unterfunktion betroffen.

##### Merke

Werden Sie bei der Interpretation der Vestibularisdiagnostik vom Nystagmusjäger zum Mustererkenner.

### Diagnostisch relevante Diskrepanzen

In einigen Fällen liefert die Diskrepanz zwischen einzelnen Untersuchungsbefunden sogar wichtige differenzialdiagnostische Hinweise.

#### Bogengangsfunktion

Bei etwa 20 % der Patient:innen werden in der Vestibularisprüfung diskordante Ergebnisse für die Funktion des horizontalen Bogengangs in der kalorischen Prüfung und im vHIT beobachtet [33, 34].

Hier sei zunächst darauf hingewiesen, dass Kalorik und vHIT zwar beide die Funktion des horizontalen Bogengangs testen, jedoch in **unterschiedlichen Frequenzbereichen**

Unterschiedliche Frequenzbereiche

(Kalorik: 0,003–0,008 Hz; vHIT: 1–6 Hz) [2]. Daher sind gewisse Unterschiede in den Ergebnissen der beiden Untersuchungen einfach testimmanent. Zudem ist zu beachten, dass es bei der **kalorischen Prüfung**

Kalorische Prüfung

deutlich mehr **Störfaktoren**

Störfaktoren

gibt, welche das Ergebnis beeinflussen können als beim vHIT, sowohl aufseiten des Untersuchten (z. B. Gehörgangsexostosen, anatomische Variationen des Gehörgangs und des Trommelfells) als auch des Untersuchers (z. B. unterschiedliche Positionierung des Wasserstrahls in beiden Ohren) [5]. Gerade bei Patient:innen mit Z. n. **Ohroperationen**

Ohroperationen

sollte daher das Ergebnis der kalorischen Prüfung vorsichtig interpretiert werden.

Besonders häufig findet sich eine Diskrepanz zwischen normalem Gain im vHIT und reduzierter kalorischer Erregbarkeit des horizontalen Bogengangs bei Vorliegen eines **Endolymphhydrops**

Endolymphhydrops.

In einer Metaanalyse von 2023 wird diese Konstellation bei 47 % (95 %-Konfidenzintervall, 95 %-KI: 37–57 %) der Patient:innen mit **M. Menière**

M. Menière

beschrieben [35]. Unterschiedliche Erklärungsmodelle hierfür werden z. B. bei McGarvie et al. [36] (fluidmechanisches Modell) und bei Shen et al. [37] (Herniation des Utrikulus in den horizontalen Bogengang) erläutert.

#### Otolithenfunktion

Während die häufigste Ursache für eine Diskrepanz zwischen reduzierten Amplituden in den **Luftleitungs-VEMPs**

Luftleitungs-VEMPs

bei normalen Resultaten in den **Knochenleitungs-VEMPs**

Knochenleitungs-VEMPs

eine Schallleitungskomponente des Mittelohrs ist, wird diese auch bisweilen bei Patient:innen mit Endolymphhydrops bzw. M. Menière beobachtet. Ähnlich wie bei der innenohrbedingten Schallleitungsschwerhörigkeit beim M. Menière wird als zugrunde liegender Mechanismus eine erhöhte Impedanz der hydropischen Endolymphräume des Innenohrs angenommen. Hieraus resultiert eine gedämpfte Schwingung der Stapesfußplatte mit einem Verlust in der Energieübertragung zwischen Mittel- und Innenohr [27].

Schließlich sei hier noch auf mögliche Diskrepanzen zwischen dem Befund der SVV und den oVEMPs eingegangen. Beide sind Parameter für die Utrikulusfunktion. Der entscheidende Unterschied ist jedoch, dass die beiden Tests sowohl anatomisch als auch physiologisch verschiedene Aspekte der Otolithenfunktion abbilden: Die **oVEMPs**

oVEMPs

messen die **dynamische Utrikulusfunktion**

Dynamische Utrikulusfunktion,

welche hauptsächlich von den vestibulären Typ-I-Haarzellen und Afferenzen mit irregulärer Ruheaktivität im Bereich der **Striola**

Striola

vermittelt wird. Im Gegensatz dazu reflektiert die **SVV**

SVV

die **statische Utrikulusfunktion**

statische Utrikulusfunktion

und bildet damit überwiegend die Integrität der vestibulären Typ-II-Haarzellen und der Afferenzen mit regulärer Ruheaktivität in der **Extrastriola**

Extrastriola

ab. Außerdem ist der Einfluss der zentral-vestibulären Kompensation auf beide Parameter bei einem persistierenden Otolithendefizit unterschiedlich: Während sich die SVV im Laufe der Kompensation normalisiert, sind die oVEMP-Antworten selbst Jahre nach einem Otolithenschaden noch reduziert/ausgefallen [12, 27, 38].

Eine Diskrepanz zwischen reduzierten/fehlenden oVEMPs und einer normalen SVV kann also (mindestens) 2 Ursachen haben: Entweder es handelt sich um eine selektive Störung im Bereich der Striola, und/oder es hat bereits eine zentrale Kompensation einer einseitigen Utrikulusunterfunktion stattgefunden.

##### Merke

Ursachen einer Diskrepanz zwischen reduzierten oVEMPs und normaler SVV können sein: selektive Störung in der Striola oder zentral kompensierte einseitige Utrikulusunterfunktion.

### Unauffällige Vestibularisprüfung

„Jetzt haben Sie auch wieder nichts gefunden.“ Diese Situation ist der Albtraum für die von Schwindel geplagten Patient:innen und ihre betreuenden Ärzt:innen: Nach wochen- bis monatelangem Warten auf den Termin, nach Durchführung der teils unangenehmen und langwierigen vestibulären Tests und nach der sorgfältigen Auswertung aller Untersuchungen finden sich keine pathologischen Befunde. Wenn Ärzt:innen in dieser Situation den Patient:innen mitteilen: „Sie haben nichts.“ oder „Mit ihrem Gleichgewicht ist alles in Ordnung.“, führt dies oft zu der oben genannten Reaktion – mit teils frustriertem, teils resigniertem, teils vorwurfsvollen Unterton. Wie lässt sich diese für alle Beteiligten **unbefriedigende Situation**

Unbefriedigende Situation

verhindern?

Entscheidend ist hier wieder die **offene Kommunikation**

Offene Kommunikation

mit den Patient:innen (s. Teil 1) und die Einordnung der apparativen Vestibularisdiagnostik in die **Gesamtschau**

Gesamtschau

aus Anamnese, klinischen und apparativen Befunden. Zudem ist die Unterscheidung zwischen strukturellen und funktionellen vestibulären Störungen wichtig. Hierfür können je nach Gesprächssituation auch Metaphern verwendet werden (z. B. „Hardware“ für ein strukturelles Defizit und „Software“ für ein funktionelles Problem). Anschauliche Beispiele für die Vermittlung dieser Zusammenhänge finden sich in einer Arbeit von Stone und Edwards sowie bei Stone [39, 40].

Folgende Möglichkeiten für eine unauffällige Vestibularisprüfung sollten in Betracht gezogen und abhängig von der Gesamtsituation mit den Patient:innen besprochen werden:

- Das vestibuläre Defizit liegt unter der **Detektionsschwelle**
  Detektionsschwelle.
  Hier lohnt sich ggf. eine Wiederholung der Tests im weiteren Verlauf.
- Es liegt ein **episodisches vestibuläres Syndrom**
  Episodisches vestibuläres Syndrom
  mit normalen Befunden im attackenfreien Intervall vor. Die positive Botschaft für die Patient:innen ist hier: „Durch die Erkrankung ist kein dauerhafter Schaden an ihrem Gleichgewichtsorgan entstanden.“ Ist die Diagnose nach der Vestibularisdiagnostik im attackenfreien Intervall noch nicht klar, sollte ein vestibuläres „event monitoring“ durchgeführt werden (Details s. Teil 1: „Dokumentation der Befunde“).
- Es liegt eine **funktionelle vestibuläre Störung**
  Funktionelle vestibuläre Störung
  vor, z. B. eine „persistent postural perceptual dizziness“ (PPPD). Hier ist es gerade das Wesen der Erkrankung, dass eine strukturelle Läsion nicht (mehr) nachweisen lässt. Das zugrunde liegende Krankheitskonzept wird sehr anschaulich erläutert bei Popkirov et al. sowie Staab [41, 42]. Generell wird empfohlen, die Begriffe „strukturell“ und „funktionell“ zu verwenden anstatt wie früher „organisch“ und „psychisch“.
  Schließlich sollte nochmals sorgfältig die Möglichkeit einer primär **nichtvestibulären Störung**
  Nichtvestibuläre Störung
  (z. B. kardiovaskuläre Problematik, Polyneuropathie) erwogen und ggf. weiter abgeklärt werden.

#### Merke

Eine normwertige Vestibularisprüfung bedeutet nicht, dass sich die Patient:innen den Schwindel nur „einbilden“.

## Fazit für die Praxis

- Erst denken, dann spülen! Fragen Sie sich vor jeder apparativen Vestibularisprüfung, welche Frage Sie genau mit welchen Untersuchungen beantworten wollen.
- Bilden Sie ein „diagnostisches Team“ mit Ihren Patient:innen und dem medizinisch-technischem Fachpersonal.
- Wählen Sie die einzelnen Tests abhängig von der Kooperationsfähigkeit und den Komorbiditäten Ihrer Patient:innen aus.
- Sprechen Sie mit Ihren Patient:innen – vor, während und nach der Vestibularisprüfung.
- Sie sind nicht allein. Nutzen Sie jede Gelegenheit, „schwierige“ Untersuchungssituationen und Befunde mit Kolleg:innen zu diskutieren. Oft haben diese schon ähnliche Erfahrungen gemacht – und im besten Fall eine Lösung gefunden.

## CME-Fragebogen

Welche Frage kann die apparative Vestibularisprüfung am ehesten beantworten?

Simuliert bzw. aggraviert der Untersuchte seine Beschwerden?

Welche Diagnose liegt bei ihm/ihr vor?

Wie sehr ist seine/ihre Lebensqualität durch die Schwindelbeschwerden eingeschränkt?

Wie gut ist seine/ihre peripher-vestibuläre Unterfunktion kompensiert?

Darf der Untersuchte in der Höhe arbeiten?

Ein bislang gesunder 50-jähriger Patient stellt sich bei Ihnen mit den typischen Symptomen eines akuten vestibulären Syndroms vor. Bei der klinischen Untersuchung fällt Ihnen auf, dass ein infantiler Nystagmus vorliegt (laut Patient seit früher Kindheit unverändert vorhanden). Welcher vestibuläre Test liefert Ihnen in dieser Situation am meisten Information?

Video-Kopfimpulstest

Drehstuhlpendelung

Vestibulär evozierte myogene Potenziale

Vibrationsinduzierter Nystagmus

Kalorische Prüfung

In der Videookulographie einer ansonsten gesunden 35-jährigen Patientin zeigt sich in mehreren Ableitungen ein dezenter Upbeat-Nystagmus. Alle anderen Befunde sind normal. Welchen diagnostischen Schritt unternehmen Sie als Erstes?

Durchführung einer Magnetresonanztomographie des Gehirns

Die Patientin fragen, ob sie raucht

Messung der subjektiven visuellen Vertikalen

Anmelden eines augenärztlichen Konsils

Messung der okulären vestibulär evozierten Potenziale

In welcher Situation erwarten Sie am ehesten eine erniedrigte Amplitude in den okulären vestibulär evozierten myogenen Potenzialen (oVEMPs) bei der Testung mit Luftleitungsstimulus?

Dehiszenz des oberen Bogenganges auf dem untersuchten Ohr

Schallleitungskomponente von 20 dB auf dem untersuchten Ohr

Sensorineurale Schwerhörigkeit von 50 dB auf dem untersuchten Ohr

Bei Tinnitus auf dem untersuchten Ohr

Neuritis vestibularis inferior des untersuchten Ohrs

Welche Folgerung können Sie am ehesten aus folgender Befundkonstellation ziehen: reduzierte oVEMP-Amplitude (okuläre vestibulär evozierte myogene Potenziale) für den rechten Utrikulus und normale subjektive visuelle Vertikale (SVV)?

Mangelnde Kooperation des Untersuchten bei den oVEMPs

Fehlende zentral-vestibuläre Kompensation eines einseitigen Utrikulusdefizits

Falsche Angaben des Untersuchten bei der SVV

Defizit der statischen Utrikulusfunktion rechts

Defizit der dynamischen Utrikulusfunktion rechts

Vor Ihnen sitzt ein 80-jähriger Patient mit einer Radikalhöhle rechts und Z. n. Versteifung der Halswirbelsäule. Daher möchte er nicht, dass ein Video-Kopfimpulstest durchgeführt wird. Welcher vestibuläre Test gibt Ihnen hier am schnellsten Auskunft über eine kompensierte einseitige Unterfunktion des rechten horizontalen Bogengangs?

Drehstuhlpendelung

Videookulographie

Vibrationsinduzierter Nystagmus

Okuläre vestibulär evozierte myogene Potenziale

Kalorische Prüfung

Welche Ursache erklärt am ehesten folgende Befundkonstellation: einseitige relative kalorische Unterfunktion rechts und normaler Befund im Video-Kopfimpulstest für alle 6 Bogengänge?

Kompensierte Unterfunktion des rechten horizontalen Bogengangs

Otholithendefizit rechts

Endolymphatischer Hydrops links

Gehörgangsexostosen rechts

Selektives Defizit der hochfrequenten Bogengangfunktion

Sie indizierten bei einer Patientin folgende vestibuläre Untersuchungen: Videookulographie (VOG, inkl. Spontan‑, Kopfschüttel- und Lagerungsnystagmus), kalorische Prüfung und subjektive visuelle Vertikale (SVV). Die Patientin äußert große Bedenken, dass sie beim Auftreten von Schwindel die Untersuchung abbrechen müsse. In welcher Reihenfolge planen Sie die Tests am ehesten?

Kalorik – SVV – VOG

VOG – SVV – Kalorik

SVV – Kalorik – VOG

SVV – VOG – Kalorik

VOG – Kalorik – SVV

Eine 45-jährige Patientin hat vor Kurzem ein Lärmtrauma erlitten. Seither leidet sie unter Hörminderung, Tinnitus und Schwindel. Welche der genannten Untersuchungen vermeiden Sie bei ihr?

VEMP (vestibulär evozierte myogene Potenziale) mit Luftleitungsstimulus

Video-Kopfimpulstest (vHIT)

VEMP mit Knochenleitungsstimulus

Otoakustische Emissionen

Vibrationsinduzierter Nystagmus

Welcher der folgenden Parameter ist ein Maß für die Kompensation einer peripher-vestibulären Störung?

Gain im Video-Kopf-Impuls-Test (vHIT)

Vorhandensein eines Spontannystagmus

VEMP-Amplitude

VEMP-Asymmetrie-Ratio

Seitendifferenz in der kalorischen Prüfung

## Abkürzungen

BPLS: Benigner paroxysmaler Lagerungsschwindel
c: Zervikal
GLP: Geschwindigkeit der langsamen Phase
HIMPs: „Head impulses“
o: Okulär
PPPD: „Persistent postural perceptual dizziness“
PROMs: „Patient-related outcome measures“
SHIMPs: „Suppression of head impulses“
SVV: Subjektive visuelle Vertikale
VEMPs: „Vestibular evoked myogenic potentials“
vHIT: Video-Kopfimpulstest
VIN: Vibrationsinduzierter Nystagmus
VOG: Videookulographie
VOR: Vestibulookulärer Reflex
